# PCMT1 regulates the migration, invasion, and apoptosis of prostate cancer through modulating the PI3K/AKT/GSK-3β pathway

**DOI:** 10.18632/aging.205152

**Published:** 2023-10-27

**Authors:** Jiacheng Zhong, Chao Yuan, Lin Liu, Yang Du, Yumin Hui, Zhiyuan Chen, Changhui Diao, Rui Yang, Guiyong Liu, Xiuheng Liu

**Affiliations:** 1 Department of Urology, Renmin Hospital of Wuhan University, Wuhan 430060, China; 2 Department of Urology, Jingzhou Central Hospital, Jingzhou 434020, China; 3 Department of Emergency, Renmin Hospital, Hubei University of Medicine, Shiyan 442000, China; 4 Department of Urology, The First People’s Hospital of Shangqiu City, Shangqiu 476100, China; 5 Department of Urology, Qianjiang Central Hospital, Qianjiang 433100, China

**Keywords:** PCMT1, prostate cancer, PI3K/AKT/GSK-3β signaling pathway

## Abstract

Protein L-isoaspartate (D-aspartate) O-methyltransferase (PCMT1) is a repair enzyme that catalyzes the conversion of isomerized aspartic acid (iso-Asp) residues into their normal structure, thereby restoring the configuration and function of proteins. Studies have shown that PCMT1 is overexpressed in several tumors and affects patients’ prognosis. However, there are few reports on the role of PCMT1 in prostate cancer (PCa). In the present research, with the assistance of The Cancer Genome Atlas Program (TCGA) database, we found that PCMT1 was overexpressed in PCa tissues. The results of quantitative reverse transcription-polymerase chain reaction (qRT-PCR), western blot and immunohistochemistry staining also showed that PCMT1 expression was significantly increased in PCa tissues and cell lines. In PCa clinical samples, PCMT1 expression was closely related to Gleason score, clinical stage, lymph node metastasis and bone metastasis. The experiments of overexpression and knockdown of PCMT1 *in vitro* or *in vivo* showed that PCMT1 can significantly promote the proliferation, migration and invasion of PCa cells, inhibit cell apoptosis, and promote the growth of PCa. We furthermore confirmed that PCMT1 regulated the migration, invasion and apoptosis of PCa cells by modulating the phosphatidylinositol 3-kinase/AKT kinase/glycogen-synthase kinase-3β (PI3K/AKT/GSK-3β) signaling pathway. Collectively, PCMT1 plays a cancer-facilitative role in PCa by promoting the proliferation, migration and invasion of PCa cells, and inhibiting apoptosis. Therefore, PCMT1 is considered to represent a novel target for treating PCa.

## INTRODUCTION

PCa ranks second among male malignancies worldwide [1]. In China, the incidence of PCa is increasing annually, and most patients with PCa are diagnosed with locally advanced or metastatic disease [2]. Despite advances in diagnosis and treatment, many patients still die of PCa every year [3]. Radical prostatectomy can be used to treat patients with localized early PCa [4]. However, most patients are diagnosed with intermediate or advanced PCa, which leads to unsatisfactory results of radical surgery or loss of opportunity for surgery [2]. For these patients, endocrine therapy is a standard treatment regimen [5]. Unfortunately, patients undergoing endocrine therapy almost inevitably progress to castration-resistant prostate cancer (CRPC) [5], for which there is currently no effective treatment. Therefore, it is necessary to explore the molecular mechanisms of PCa occurrence and development and discover more effective biomarkers, so as to provide new therapeutic targets for PCa therapy.

Under some stress-related conditions such as hypoxic stress and sunburn, L-aspartic acid (L-Asp) and L-asparagine (L-Asn) residues of some proteins undergo non-enzymatic modification to become isomerized aspartic acid (iso-Asp) residues, which can result in structurally nonfunctional proteins [6]. PCMT1 is a repair enzyme that widely exists in the human body and can initiate the conversion of iso-Asp residues to their normal configuration, thereby restoring the structure and function of those damaged proteins [6]. PCMT1 catalyzes the transfer of the methyl group of S-adenosylmethionine (SAM) to the iso-Asp residue, thereby converting it to L-Asp [7]. In lung adenocarcinoma, the expression of PCMT1 in patients with stage I or pre-invasive lesions is significantly lower than that in patients with stage II-IV or invasive adenocarcinoma. Patients with high PCMT1 expression have a shorter survival period, and high PCMT1 expression is an independent unfavorable prognostic factor [8]. PCMT1 has also confirmed to be an unfavorable prognostic factor in bladder cancer. The expression level of PCMT1 in bladder cancer cells and tissues is increased significantly compared to normal uroepithelial cells and tissues. PCMT1 expression is closely related to clinical grade, muscle infiltration, lymph node metastasis and distant metastasis of bladder cancer patients [9]. At present, the role of PCMT1 in PCa is still unknown.

In this study, we examined the expression of PCMT1 in PCa tissues and cell lines. Furthermore, we analyzed the correlation between PCMT1 expression and the clinicopathological characteristics of PCa patients. In addition, we explored the biological role of PCMT1 in PCa cell proliferation, invasion, migration and apoptosis and its possible mechanisms.

## RESULTS

### Expression of PCMT1 in PCa tissues and cell lines

**Figure 1:**
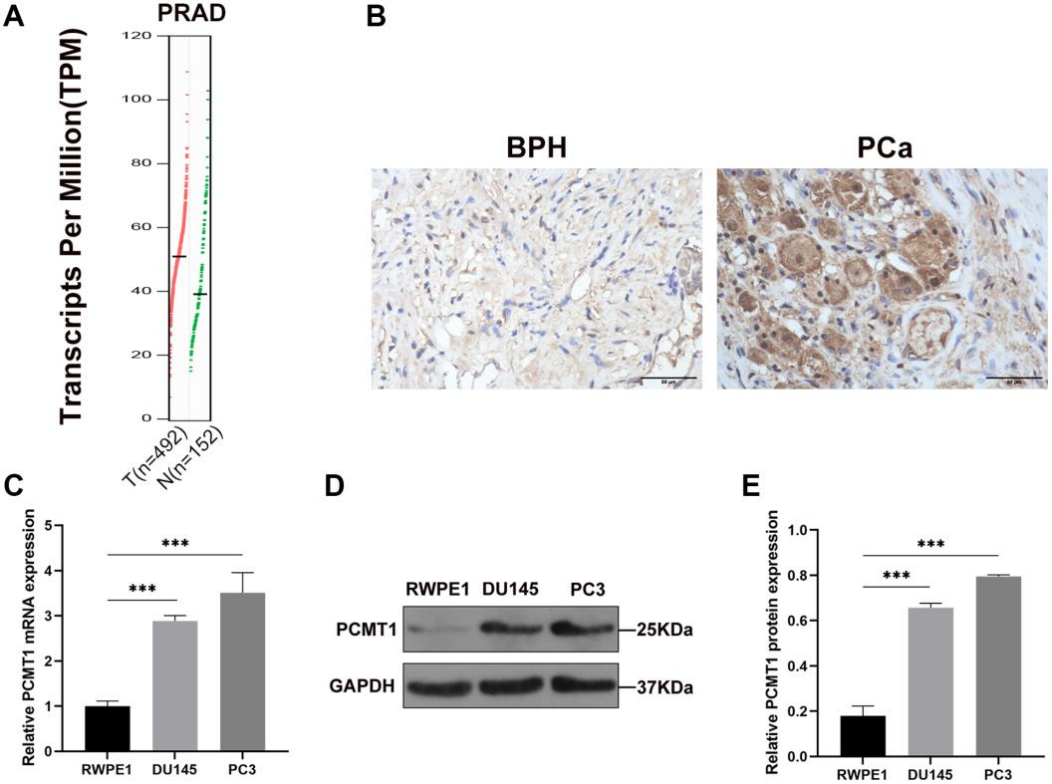
**Expression of PCMT1 in PCa tissues and cell lines.** (**A**) The expression of PCMT1 was identified in 492 PCa and 152 normal prostate tissues from TCGA and GTEx data. (**B**) PCMT1 protein expression in BPH and PCa tissues (IHC, ×400). (**C**) PCMT1 mRNA expression in PCa cell lines and the prostate epithelial cell line. (**D**) PCMT1 protein expression in PCa cell lines and normal prostate epithelial cell line. (**E**) Quantitative analysis of PCMT1 protein expression. Data are expressed as mean ± SD of at least three experiments. ^*^P < 0.05 and ^***^P < 0.001.

At first, we used GEPIA database to analyze the differential expressions of PCMT1 in PCa samples and normal prostate samples. The GEPIA database by matching TCGA and GTEx data showed that the PCMT1 expression was upregulated in PCa samples compared to normal prostate samples (*P* < 0.05, Figure 1A). And then, we detected PCMT1 protein expression in 78 PCa and 45 BPH samples by immunohistochemistry (IHC) staining (Figure 1B). The results showed that PCMT1 protein expression in PCa tissues was higher than that in BPH tissues (*P* = 0.016, Table 1).

**Table 1:** PCMT1 protein expression in PCa tissues and BPH tissues.

**Group**	***n***	**PCMT1 protein expression**		***P***
		**Positive**	**Negative**	
PCa	78	47 (60.3%)	31 (39.7%)	0.016
BPH	45	17 (37.8%)	28 (62.2%)

Furthermore, we detected the expression of PCMT1 mRNA and protein in PCa cells. The results showed that the expression of PCMT1 mRNA in both PCa cell lines (PC3 and DU145) was significantly upregulated compared with normal prostate epithelial cell line (RWPE1) (Figure 1C). Meanwhile, both PCa cell lines had significantly higher PCMT1 protein expression than the RWPE1 cell line (Figure 1D, 1E).

Overall, these results demonstrated that PCMT1 is upregulated in both PCa tissues and cell lines.

### Correlation between PCMT1 protein expression and clinicopathological characteristics of patients with PCa

As shown in Table 2, PCMT1 protein expression was closely related to Gleason score, clinical stage, lymph node metastasis and bone metastasis (*P* = 0.003, 0.003, 0.036, 0.020, respectively) of patients with PCa, but was not associated with age (*P* = 0.053) and preoperative prostate specific antigen (PSA) (*P* = 0.679).

**Table 2:** Association between PCMT1 protein expression and clinicopathological characteristics in patients with PCa.

**Characteristics**	***n***	**PCMT1 protein expression**		**χ^2^**	***P***
		**Positive**	**Negative**		
Age (y)					
<65	30	14	16	3.759	0.053
≥65	48	33	15		
Preoperative PSA (ng/mL)					
≤10	33	19	14	0.172	0.679
>10	45	28	17		
Gleason score					
≤7	39	17	22	9.047	0.003
>7	39	30	9		
Clinical stage					
T_1_+T_2_	36	15	21	9.648	0.003
T_3_+T_4_	42	32	10		
Lymph node metastasis					
Without	55	29	26	4.415	0.036
With	23	18	5		
Bone metastasis					
Without	63	34	29	5.409	0.020
With	15	13	2		

### Effect of PCMT1 on the proliferation of PCa cells

**Figure 2:**
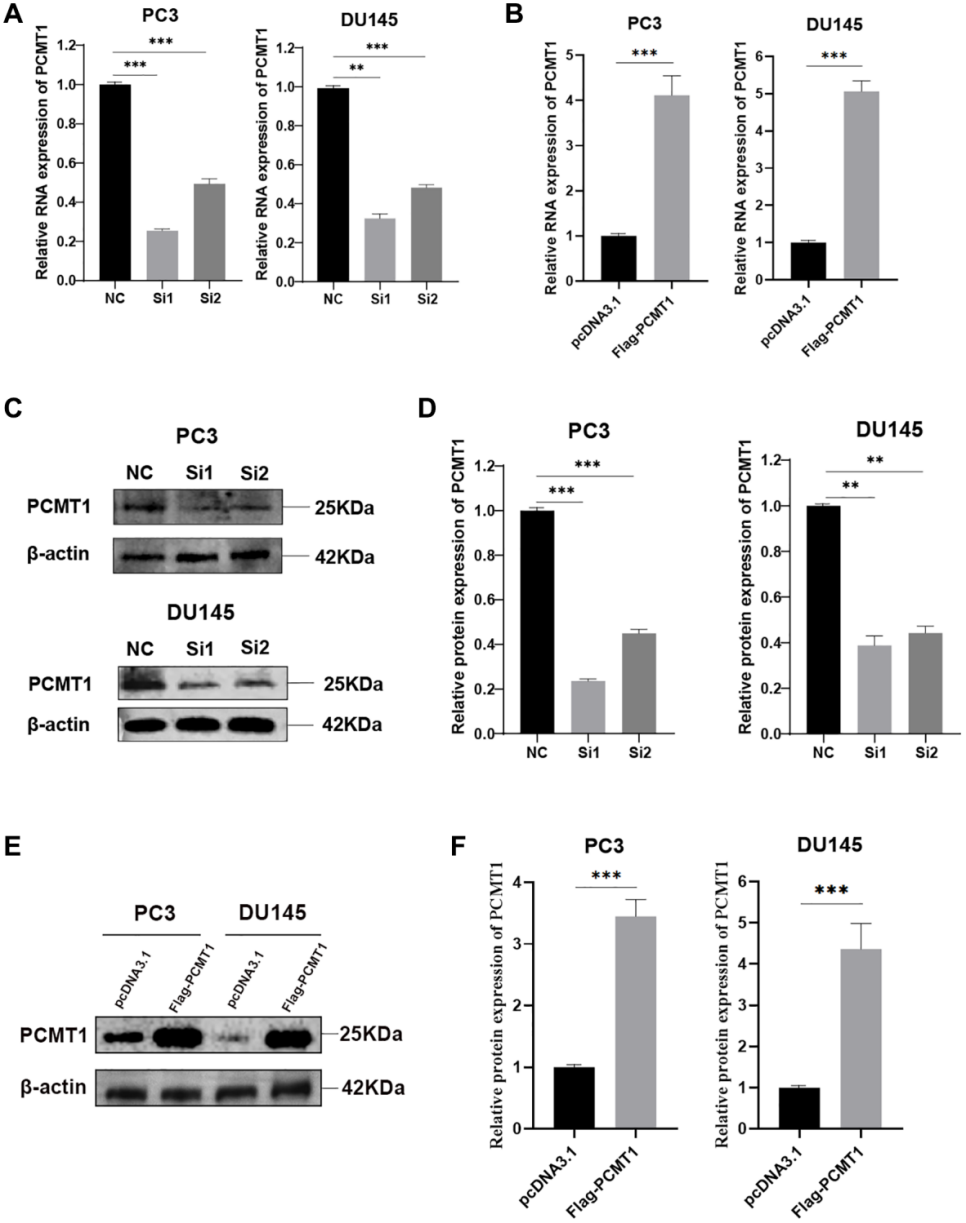
**PC3 and DU145 cells transfected with siRNA-PCMT1 down-regulated PCMT1 expression and Flag-PCMT1 up-regulated PCMT1 expression.** (**A**, **B**) siRNA-PCMT1 transfection down-regulated PCMT1 mRNA expression of PC3 and DU145 cells, respectively. (**C**, **D**) siRNA-PCMT1 transfection down-regulated PCMT1 protein expression of PC3 and DU145 cells. (**E**, **F**) Flag-PCMT1 transfection up-regulated PCMT1 protein expression of PC3 and DU145 cells. Data are presented as mean ± SD of at least three experiments. ^**^P < 0.01 and ^***^P < 0.001.

To explore the potential effect of PCMT1 on PCa cells, we used siRNAs and plasmids for cell transfection. Two different siRNAs targeting PCMT1 were used to inhibit PCMT1 expression in PCa cells, meanwhile, plasmids were used as vectors to enhance the expression of PCMT1 in PCa cells. qRT-PCR and western blot were used to detect the effect of transfection, which showed that PCMT1 mRNA and protein were both significantly down-regulated by these two kinds of siRNA in PC3 and DU145 cell lines (Figure 2A, 2C, 2D). Given the better knockdown effect of the first siRNA, we chose it to carry out the follow-up experiment. Meanwhile, PCMT1 mRNA and protein were triumphantly enhanced in the two cell lines by plasmid transfection (Figure 2B, 2E, 2F).

**Figure 3:**
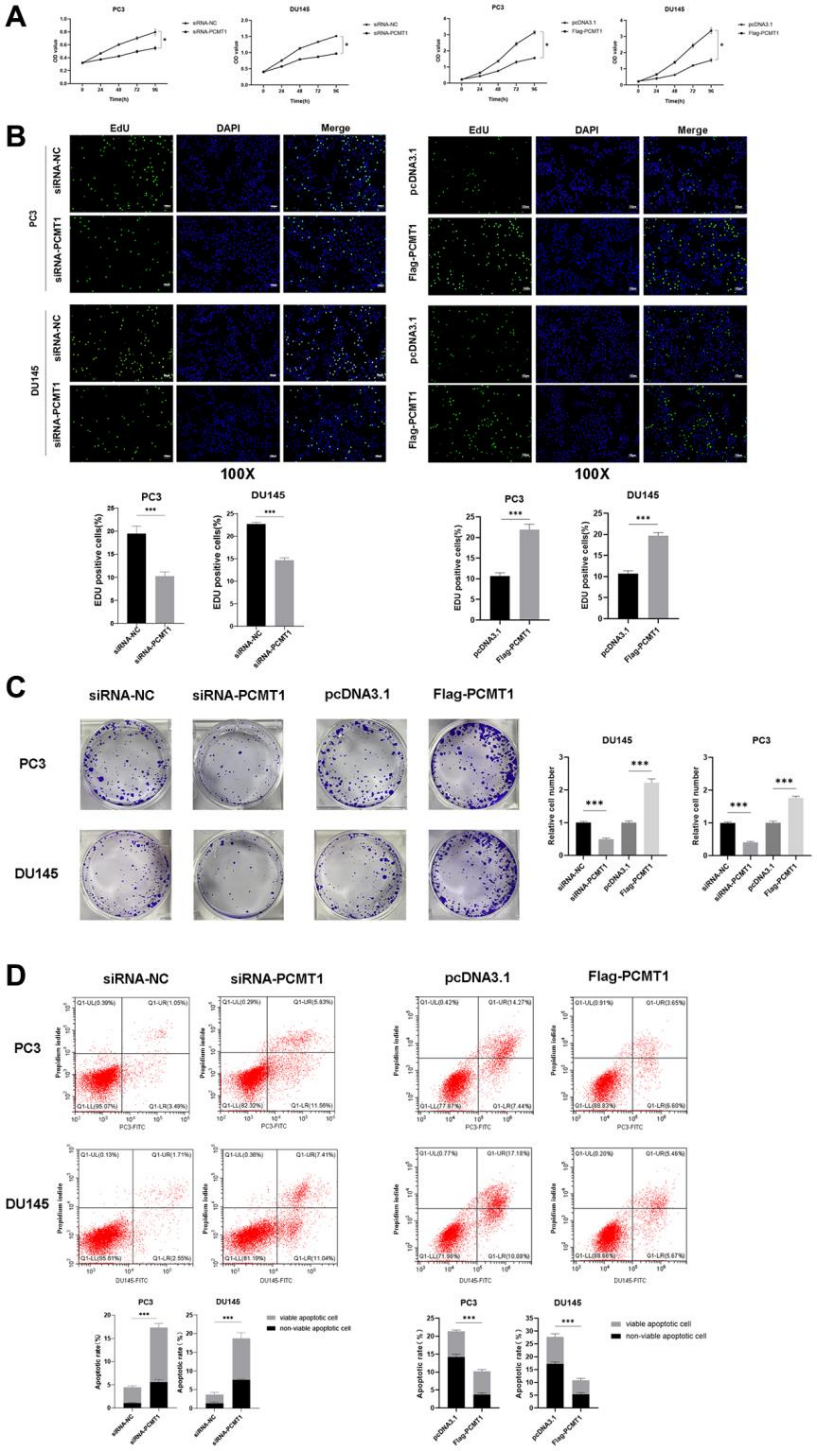
**Impact of PCMT1 inhibition on the proliferation and apoptosis of PCa cells.** (**A**, **B**) Effect of PCMT1 inhibition and overexpression on PCa cell proliferation measured by a CCK-8 assay or EdU assay. (**C**) Effect of PCMT1 inhibition and overexpression on PCa cell proliferation measured by a colony formation experiment. (**D**) Effect of PCMT1 inhibition and overexpression on PCa cell apoptosis measured by a flow cytometry assay. Data are expressed as mean ± SD of at least three experiments. ^***^P < 0.001.

We first investigated the effect of PCMT1 on the proliferation of PCa cells. As shown in Figure 3A, treatment of PCMT1 knockdown with siRNA prominently suppressed cell proliferation curve obtained by the CCK-8 assay (*P* < 0.05, Figure 3A), comparing with the negative-control group, whereas, PCMT1 expression significantly promote the curve. In addition, the results of both the EdU assay and the colony formation assay verified that the proliferation of PCa cells was inhibited by PCMT1 knockdown and reinforced by PCMT1 overexpression (Figure 3B–3C).

### Effect of PCMT1 on the apoptosis of PCa cells

The flow cytometry was performed to detect the effect of PCMT1 on the apoptosis of PCa cells. As shown in Figure 3D, the apoptosis of PCa cells was significantly promoted by PCMT1 inhibition and notably suppressed by PCMT1 overexpression. (*P* < 0.001, Figure 3D).

### Effect of PCMT1 on the migration and invasion of PCa cells

**Figure 4:**
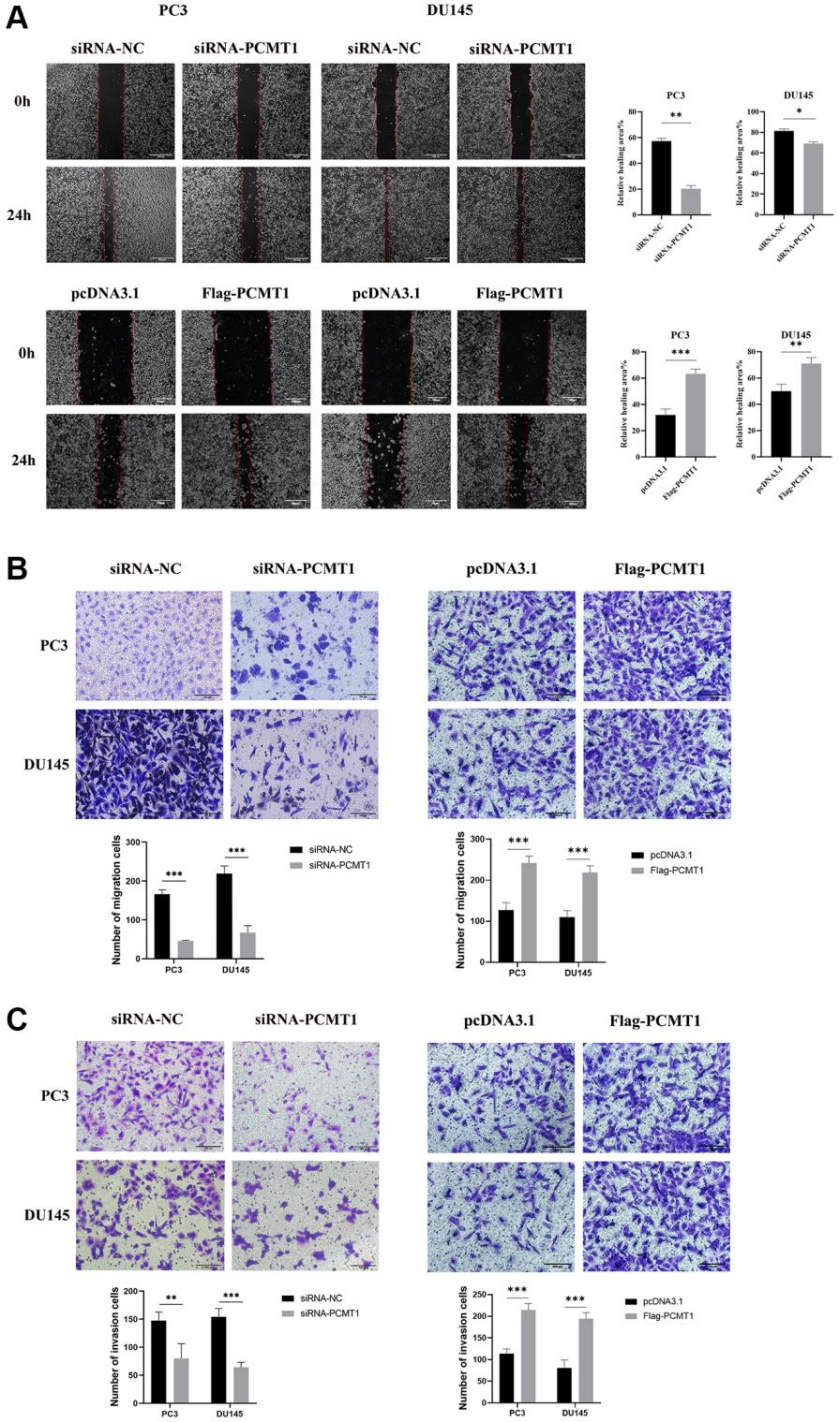
**Impact of PCMT1 inhibition on the migration and invasion of PCa cell lines.** (**A**) Effect of PCMT1 inhibition on PCa cell migration measured by wound-healing assay (×40). (**B**) PCa cell migration ability was assessed by a transwell migration assay (×200). (**C**) PCa cell invasion ability was examined by a transwell invasion assay (×200). Data are expressed as mean ± SD of at least three experiments. ^*^P < 0.05, ^**^P < 0.01, ^***^P < 0.001.

The effects of PCMT1 on the migration and invasion of PCa cells were studied using wound-healing assay and transwell assays. The results showed that the migration ability of PCa cells was significantly inhibited by down-regulating PCMT1 expression and dramatically reinforced by up-regulating PCMT1 (*P* < 0.05, Figure 4A, 4B). The results of the transwell invasion experiment showed that the invasion ability of PCa cells was significantly inhibited by down-regulating PCMT1 expression and prominently heightened by up-regulating PCMT1 (*P* < 0.001, Figure 4C).

### PCMT1 regulates PCa progression by modulating the PI3K/AKT/GSK-3β signaling pathway

**Figure 5:**
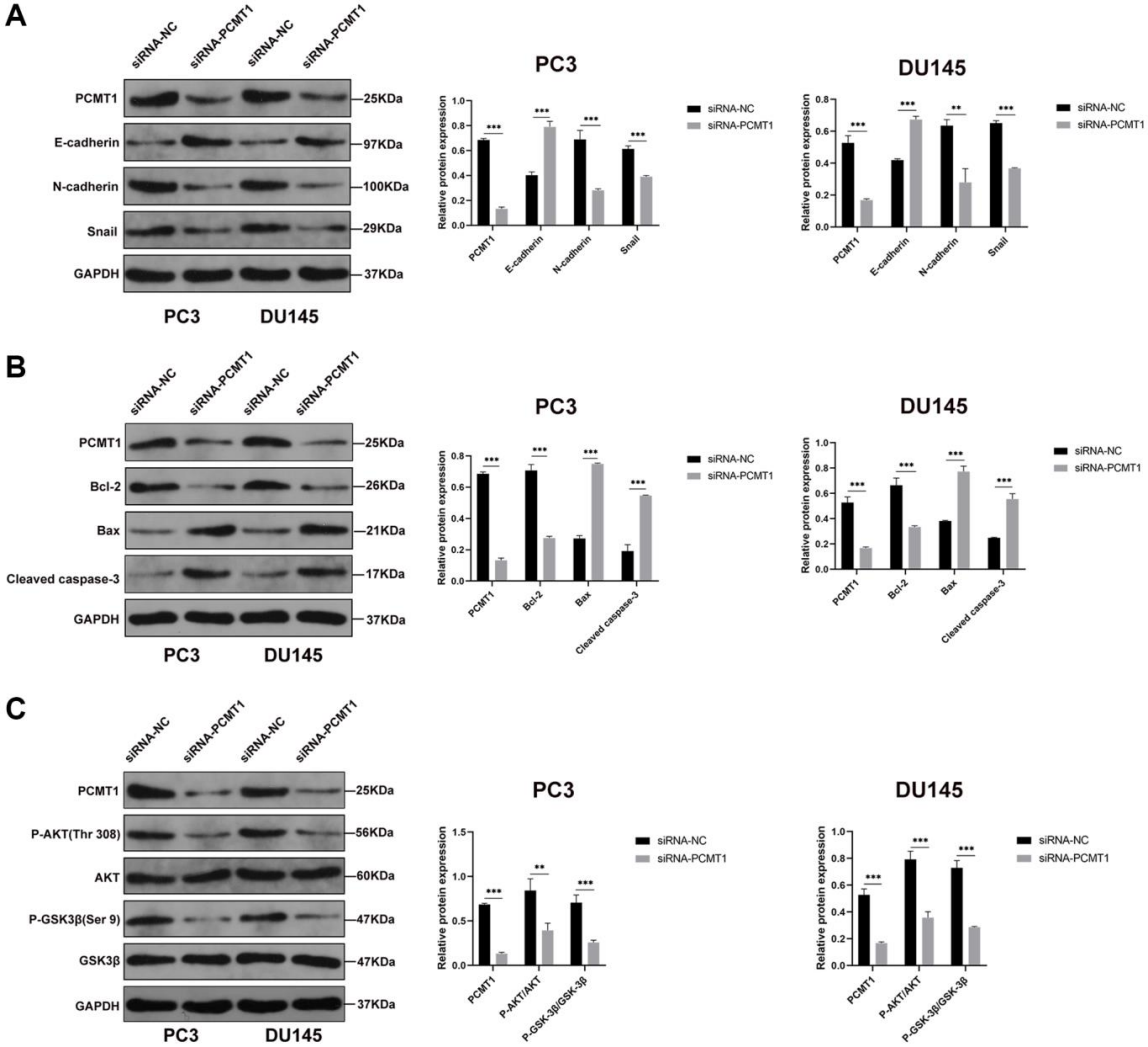
**PCMT1 regulates the EMT and apoptosis of PCa cells by modulating the PI3K/AKT/GSK-β signaling pathway.** (**A**) Protein expression of factors involved in EMT, and quantification of the protein levels. (**B**) Protein expression of factors involved in apoptosis, and quantification of the protein levels. (**C**) Protein expression of P-AKT, AKT, P-GSK-3β, and GSK-3β, and quantification of the protein levels. Data are expressed as mean ± SD of at least three experiments. ^**^P < 0. 01, ^***^P < 0. 001.

To further explore the molecular mechanism underlying the effects of PCMT1 on the malignant phenotypes of PCa cells, we detected the expression of several molecules related to migration, invasion and apoptosis by western blot. The results showed that PCMT1 inhibition could obviously increase the expression level of E-cadherin and decrease the expression of N-cadherin and Snail (Figure 5A). It was revealed that PCMT1 inhibition suppressed the migration and invasion of PCa cells by regulating the expression of epithelial mesenchymal transition (EMT)-related genes. Additionally, PCMT1 inhibition promoted the expression of Bax (a pro-apoptotic molecule) and cleaved caspase-3 (the most important terminal splicing enzyme in the process of apoptosis), and suppressed Bcl-2 (an anti-apoptotic molecule) expression (Figure 5B), thereby promoting the apoptosis of PCa cells. Published studies have shown that the PI3K/AKT/GSK-3β pathway played an important role in the process of tumor cells migration, invasion, and apoptosis [10, 11]. To explore whether PCMT1 regulates the metastasis and apoptosis of PCa cells through the PI3K/AKT/GSK-3β signaling pathway, we detected the expression of related proteins. The results showed that PCMT1 inhibition decreased the levels of P-AKT and P-GSK-3β (Figure 5C), suggesting that PCMT1 may regulate PCa progression by modulating the PI3K/AKT/GSK-3β signaling pathway.

**Figure 6:**
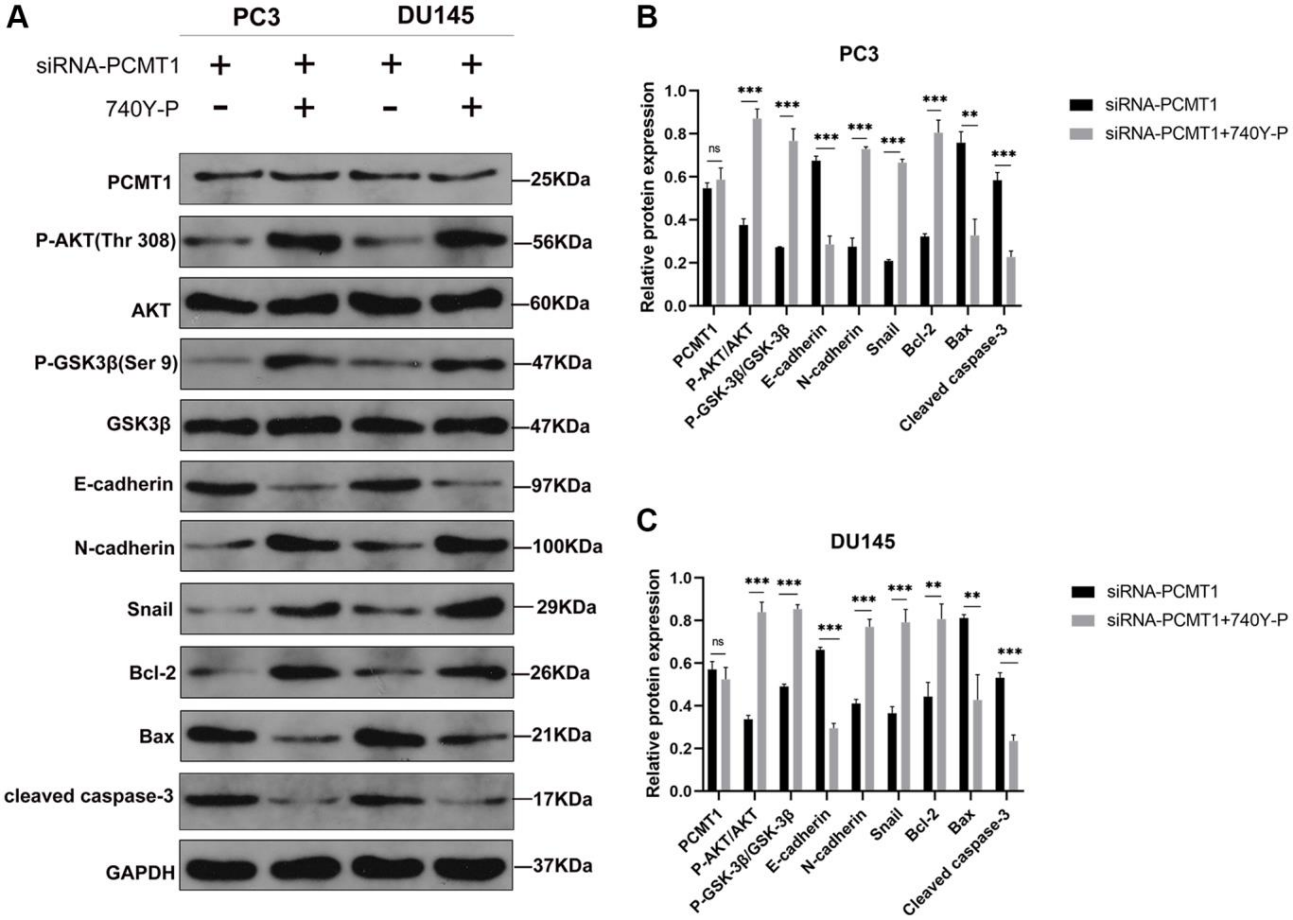
**PI3K/AKT signaling pathway activator 740Y-P affects the protein expression of related factors in siRNA-PCMT1 PCa cells.** (**A**) Protein expression of E-cadherin, N-cadherin, Snail, Bax, Bcl-2, cleaved caspase-3, P-AKT, AKT, P-GSK-3β, and GSK-3β; in PC3 and DU145 cell lines. (**B**, **C**) Quantitative analysis of the protein expression levels in PC3 and DU145 cell lines. Data are expressed as mean ± SD of at least three experiments. ^**^P < 0. 01, ^***^P < 0. 001. Abbreviation: n. s: not significant.

To further verify the correlation between PCMT1 and PI3K/AKT/GSK-3β signaling pathway, we next applied 740-YP, a PI3K/AKT signaling pathway activator, to the siRNA-PCMT1 cells, which was used to reverse the effects of siRNA-PCMT1. The results of western blot showed that the expression levels of P-AKT, P-GSK-3β, Snail, N-cadherin, and Bcl-2 were increased, while the expression levels of E-cadherin, Bax and cleaved caspase-3 were decreased after treatment with 740Y-P in the siRNA-PCMT1 PC3 and DU145 cells (Figure 6A–6C). Interestingly, the expression of PCMT1 was not affected, suggesting that PCMT1 may be an upstream molecule of PI3K/AKT/GSK-3β signaling pathway.

**Figure 7:**
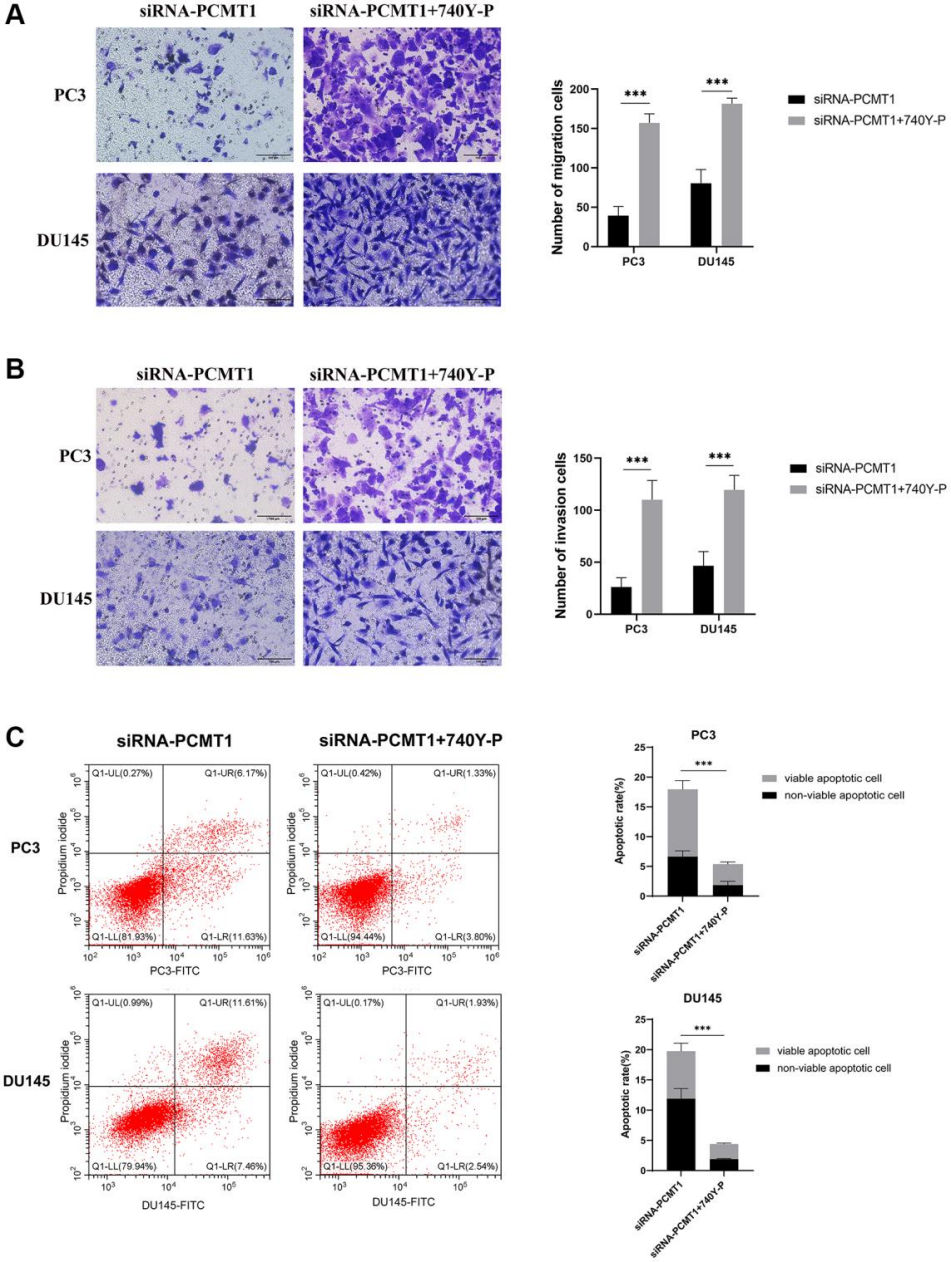
**PI3K activator 740Y-P can reverse the effect of siRNA-PCMT1 on the migration, invasion, and apoptosis of PCa cell lines.** (**A**) The migration ability of PCa cells was measured by a transwell migration assay (×200). (**B**) The invasion ability of PCa cells was measured by a transwell invasion assay (×200). (**C**) The apoptotic rate of PCa cells was measured by a flow cytomet assay. Data are expressed as mean ± SD of at least three experiments. ^***^P < 0. 001.

Transwell migration and invasion assays showed that the cell migration and invasion abilities of siRNA-PCMT1 PC3 and DU145 cells were enhanced after treatment with 740Y-P (Figure 7A, 7B). The results of the apoptosis assay showed that the number of apoptotic cells was decreased in siRNA-PCMT1 PC3 and DU145 cells after treatment with 740Y-P (Figure 7C). Together, these results indicate that PCMT1 regulates PCa progression by modulating the PI3K/AKT/GSK-3β signaling pathway.

### Overexpression or knockdown of PCMT1 influences the growth and progression of PCa *in vivo*

**Figure 8:**
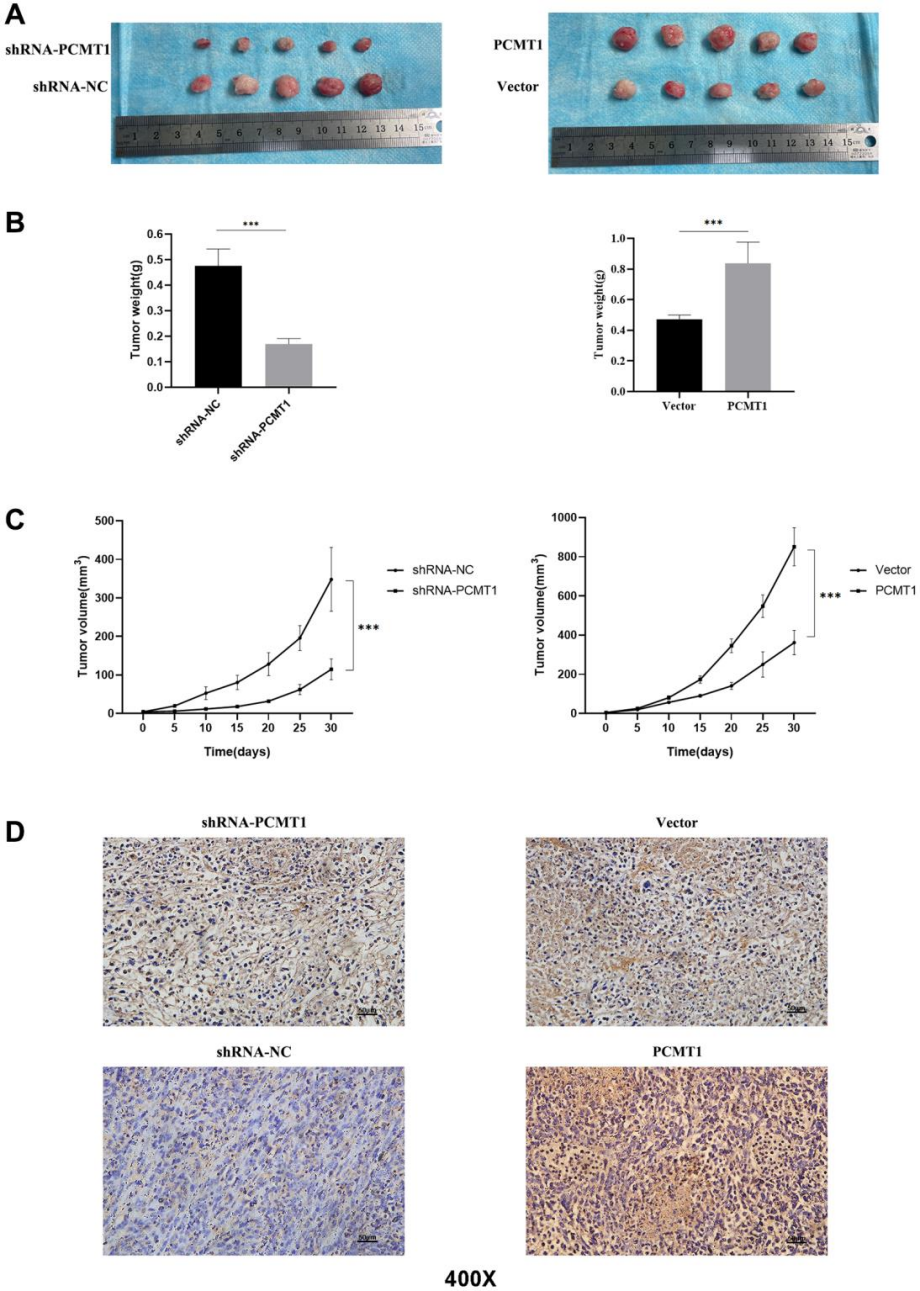
**PCMT1 can affect the proliferation of prostate cancer cells *in vivo*.** (**A**) Comparison of tumor tissue size in different groups of nude mice. (**B**) Comparison of tumor weights in different groups of nude mice. (**C**) The tumor volume was calculated at each time point. (**D**) PCMT1 protein expression in nude mice tumor tissues (IHC, ×200). Data are expressed as mean ± SD of at least three experiments. ^***^P < 0. 001.

In addition, in order to further verify the role of PCMT1 *in vivo*, PCa cells transfected steadily by lentivirus with overexpression or knockdown of PCMT1 were injected into nude mice. The results showed that, compared with the control group, tumor size, weight and growth rate in the PCMT1 overexpression group were significantly increased, while those in the knockdown group were significantly decreased (Figure 8A–8C). At the same time, we verified by immunohistochemistry that the expression of PCMT1 decreased in shRNA-PCMT1 group and increased in PCMT1 group (Figure 8D). In conclusion, overexpression of PCMT1 in PCa cells promotes tumor growth *in vivo*.

## DISCUSSION

The human PCMT1 gene is located on chromosome 6p22.3-6q24, with a total length of approximately 60 kb, containing 8 exons and 7 introns [12]. The protein encoded by PCMT1 gene is a protein methyltransferase that exists widely in the human body with a molecular weight of 24.5 kDa [13]. PCMT1 protein generally exists in the cytoplasm as a monomer and consists of two isoforms produced by alternative splicing [13].

PCMT1 protein contains an RNA binding domain, a methyltransferase domain, and a S-adenosylme- thionine (SAM) binding domain [14]. PCMT1 catalyzes the methyl groups of SAM to the side chain carboxyl groups of L-isoaspartic acid and D-aspartic acid residues, so that the protein can restore normal conformation and function [15]. PCMT1 also participates in biological processes including synaptic transmission, cell matrix interaction and apoptosis through the deamidation of asparagine [16, 17].

Published studies on PCMT1 are mainly concentrated in non-neoplastic disease. Some studies have reported that PCMT1 is closely related to the occurrence of several neurological diseases, including Alzheimer’s disease, Parkinson’s disease, and multiple sclerosis [18–20]. At present, there are relatively few studies on PCMT1 in the field of oncology, but the potential value of PCMT1 in neoplastic diseases can be found by summarizing the existing research results. PCMT1 overexpression may be an important factor that allows tumor cells to survive longer than normal cells [9]. Until now, there has been no relevant research on the relationship between PCMT1 expression and PCa. Our studies confirmed that PCMT1 was overexpressed in PCa cells and tissues, and we found PCMT1 expression was closely associated with Gleason score, clinical stage, lymph node metastasis, and bone metastasis of patients with PCa. To explore the biological functions of PCMT1 on PCa cells, we down-regulated the expression of PCMT1 by siRNA transfection. The results showed that PCMT1 inhibition significantly suppressed the proliferation, migration and invasion of PCa cells, and promoted apoptosis. These results show that PCMT1 plays an important role in facilitating PCa occurrence and development. In order to further verify the function of PCMT1 *in vivo*, tumor formation experiments in nude mice were conducted to prove that PCMT1 also promotes tumor growth *in vivo*.

EMT is a process by which epithelial cells obtain a mesenchymal cell phenotype after a specific transformation procedure, and is an important mechanism for malignant transformation during tumor development [21]. Tumor cells that have undergone EMT show loss of epithelial cell phenotype, and are typically characterized by decreased expression of E-cadherin [22]. In the process of tumor development, the down-regulation of E-cadherin expression is mainly initiated by Snail/Slug family [23].

The PI3K/AKT pathway is an important signaling pathway in the human body, and increasing studies have shown that it is closely related to the survival of tumor cells [24]. GSK-3β, as a main intracellular serine/threonine family kinase, is an important substrate of AKT [25]. The PI3K/AKT/GSK-3β signaling pathway is one of the main pathways involved in the regulation of tumor cell EMT [26, 27]. GSK-3β maintains an activated state in epithelial cells, and plays a critical role in maintaining the epithelial cell framework. GSK-3β can inhibit the expression level and transcription activity of Snail by suppressing the nuclear translocation and transcription activity of Snail, and promoting degradation [23]. The activity of GSK-3β is regulated by different phosphorylation modes, among which the phosphorylation at Ser9 can inhibit its kinase activity [28, 29]. P-AKT phosphorylates GSK-3β at Ser9, which up-regulates of the transcriptional activity and expression level of Snail [26]. The increase in Snail expression level inhibits the transcriptional activity of E-cadherin and down-regulates its expression, thereby inducing tumor cell EMT and promoting metastasis [23].

**Figure 9:**
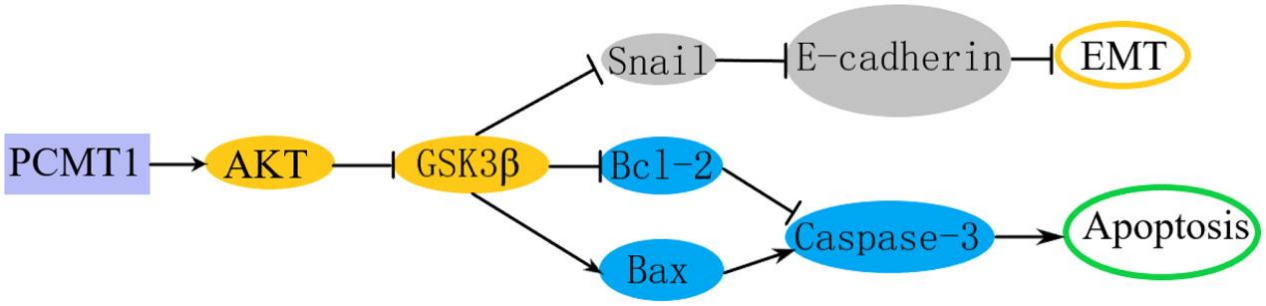
Schematic representation of the mechanism through which PCMT1 regulated EMT and apoptosis in PCa cells via PI3K/AKT/GSK-3β pathway.

To explore whether PCMT1 regulates the EMT of PCa through PI3K/AKT/GSK-3β signaling pathway, we detected Snail, p-GSK-3β (Ser9) and GSK-3β from protein level by western blot. The results showed that the down-regulation of PCMT1 was related to the decrease of Snail expression. Meanwhile, the expression level of p-GSK-3β (Ser9) was also significantly decreased. Therefore, down-regulation of PCMT1 could inhibit the Ser9 phosphorylation of GSK-3β, promote the degradation of Snail, and then reduce the transcriptional inhibition of E-cadherin, thereby inhibiting the migration and invasion of PCa cells (Figure 9).

The PI3K/AKT/GSK-3β pathway also plays a crucial role in regulating tumor cell apoptosis [30]. P-GSK-3β inhibits the mitochondrial translocation of the pro-apoptotic molecule Bax and the degradation of the anti-apoptotic molecule Bcl-2 to prevent apoptosis [30, 31]. To explore the molecular mechanism by which PCMT1 regulates PCa cell apoptosis, we tested the protein levels related to cell apoptosis by western blot. We found that down-regulating PCMT1 obviously inhibited the expression of Bcl-2 and increased the expression of Bax and activated caspase-3. Watcharasit et al. demonstrated that the tumor suppressor p53 could directly bind to GSK-3β to activate GSK-3β, which increased the expression level of activated caspase-3, thereby promoting tumor cell apoptosis [32]. PCMT1 inhibition may regulate the expression of apoptosis-related proteins by modulating the PI3K/AKT/ GSK-3β pathway, thereby promoting PCa cell apoptosis (Figure 9). Furthermore, 740Y-P, reactivating PI3K/AKT signaling pathway, was utilized to indicate that PCMT1 primarily affected EMT and Apoptosis through the AKT pathway.

Epigenetics refer to the regulation of gene expression by non-genetic changes, such as DNA methylation and histone modification. This regulation is heritable and does not depend on changes in DNA sequence [33]. Epigenetic mechanisms lead to genome instability and improper gene expression, which are the most typical changes in PCa [34]. Epigenetic mechanisms, including specific hypermethylation and histone modifications, play a vital role in the progression of PCa [35].

DNA methylation refers to the addition of a methyl group to the 5′- carbon of cytosine in CpG islands and is catalyzed by DNA methyltransferases. CpG islands are regions rich in CpG dinucleotides, usually located near the promoters of many genes. Therefore, methylation of CpG islands in the regions of gene promoters can lead to transcriptional inhibition, thereby affecting gene expression [36]. DNA methylation has been gradually discovered and recognized in many human tumors including PCa [34, 37]. PTEN gene is the main inhibitor of the PI3K/AKT signaling pathway, and the loss of PTEN function up-regulates P-AKT expression and affects the expression of downstream molecules. Recent studies have shown that the inactivation of PTEN gene is related to the hypermethylation status of its promoter [38]. Keil et al. discovered that PCa cell invasion and progression are associated with hypermethylation of the E-cadherin-1 gene promoter [39]. Therefore, PCMT1 might promote the progression of PCa by up-regulating the methylation of related genes.

Eukaryotic DNA is tightly surrounded by nucleosomes composed of histones, and many sites on the histones can be modified. Histone modification can affect the affinity between histones and DNA double-strand, thereby changing the looseness and aggregation state of chromatin, which in turn affects the binding of transcription factors and other regulatory proteins to chromatin, and affects gene expression [40]. Due to their long half-life and post-translational modifications, histones can also accumulate spontaneous chemical changes, which affect their function and require protein repair or degradation. One of the main sources of such protein damage or aging is the conversion of aspartic acid to isoaspartic acid residues, which can be repaired by PCMT1, thereby restoring the function of histones [41]. PCMT1 may be involved in the repair of damaged histones in tumor cells, thereby inhibiting tumor cell apoptosis.

## CONCLUSION

In summary, our results showed that PCMT1 expression was significantly unregulated in PCa tissues and cell lines, and the expression of PCMT1 was obviously related to Gleason score, clinical stage, lymph node metastasis and bone metastasis of patients with PCa. PCMT1 inhibition suppressed the proliferation, migration, and invasion of PCa cells, and promoted apoptosis. Besides, our study suggested that PCMT1 might regulate the migration, invasion and apoptosis of PCa cells by modulating the PI3K/AKT/GSK-3β signaling pathway and also can promote tumor growth in the *in vivo*. Overall, PCMT1 may function as an oncogene in PCa, and therefore, may represent a novel biomarker in the clinical treatment of PCa.

## MATERIALS AND METHODS

### Tissue specimens

We obtained tissue paraffin sections from 78 patients with PCa and 45 patients with benign prostatic hyperplasia (BPH) from the Department of Pathology, Renmin Hospital of Wuhan University. These patients had undergone resection in the Department of Urology, Renmin Hospital of Wuhan University from 2019 to 2021. All patients were diagnosed by the pathologists, and none of them had received preoperative neoadjuvant treatment before surgery. The clinical medical records of these PCa patients were collected. The collection of tissue specimens and experimental methods were approved by the Ethics Committee of Renmin Hospital of Wuhan University, and informed consent was obtained from all patients.

### Immunohistochemistry staining

The paraffin-embedded sections were deparaffinized and rehydrated. Citrate buffer (pH 6.0) was used for antigen retrieval, followed by 3% H_2_O_2_ for 15 min to eliminate endogenous peroxidase activity. The sections were incubated overnight with PCMT1 antibody (Proteintech, Wuhan, China, dilution 1:100) at 4°C. After adding the secondary antibody, the sections were reacted with 3, 3′-diaminobenzidine (DAB) for 2 min, washed with tap water, and then stained with hematoxylin. Finally, the sections were observed and photographed under a microscope.

### Evaluation of immunostaining

The immunostaining results were independently scored by two pathologists who did not know the clinical parameters. Staining was evaluated on the basis of the proportion and intensity of positively stained cells. The proportion of positively stained cells ranges from 0% to 100%, and the staining intensity varies from weak to strong. The staining intensity and range scores were determined as follows: (intensity) negative = 0; weak = 1; moderate = 2; strong = 3, and (range) 0% = 0; 1–10% = 1; 10–50% = 2; >50% = 3 [42]. The final score was calculated by multiplying the staining intensity score and the staining range score. Negative expression of PCMT1 was defined as 0 to 4 points, and positive expression of PCMT1 was defined as ≥4 points.

### Cell culture and transfection

PC3, DU145, and RWPE-1 cells were purchased from Procell (Wuhan, China) and maintained in RPMI-1640 medium with 10% fetal bovine serum and 1% penicillin-streptomycin, and were routinely cultured and subcultured in a 37°C, 5% CO_2_ incubator. Small interfering RNAs (siRNAs) specific for PCMT1 (siRNA-1: sense, 5′-GAGCAGUAUGACAAGCUA CAATT-3′; antisense, 5′-UUGUAGCUUGUCAUAC UGCUCTT-3′ and siRNA-2: sense, 5′-CAGUA UGACAAGCUACAAGAUTT-3′; antisense, 5′-AUCU UGUAGCUUGUCAUACUGTT-3′) (Sangon Biotech, Shanghai, China) were used to transfect PC3 and DU145 cells, which express relatively high levels of PCMT1. The negative control siRNA served as a control nontargeting siRNA (N.C.). PC3 and DU145 cells in the logarithmic growth phase were seeded into 6-cm dishes containing 5 mL of RPMI-1640 with 10% FBS. After the density of the cells reached nearly 75% within 24 hours, the cells were transfected with 600 pmol of siRNA-NC or siRNA-1 and siRNA-2 in the presence of 24 μL of Lipo 8000^™^ Transfection Reagent (Beyotime Biotechnology, Shanghai, China). After 48 hours of incubation following siRNA transfection, total RNA and protein were extracted. For the overexpressed PCMT1, full-length PCMT1 was connected to the pcDNA3.1 plasmid (Sangon Biotech) and the empty vector is regarded as NC. Cells were transfected with indicated plasmids by using Lipofectamine 8000 mentioned above as recommended by the product protocol. Otherwise, we used 50 μg/mL 740Y-P (Cayman, USA) to treat the cells to rescue the activity of the PI3K/AKT signaling pathway. To steadily knock down PCMT1, we also constructed plasmids by using lentiviral vector pLKO.1 and the short hairpin RNAs (shRNA) (sense, 5′-CCAGGCGCTAATAGATCA GTT-3′; antisense, 5′-AACTGATCTATTAGCGC CTGG-3′) that has been designed. In the meantime, in order to statically express PCMT1 in PCa cells, PCR was used to clone PCMT1 into pCDH-puro lentivirus vector. Next, we cotransfected them into 293T cells with helper plasmid psPAX2 and pMD2. G. Twenty-four hours after transfection, fresh medium with 10% fetal bovine serum was used to replace the old medium. After another twenty-four hours, culture medium without 293T cells was mixed with culture medium for PCa cells. After 48h, shRNA-positive PCa cells were screened with puromycin [43].

### qRT-PCR

Total RNA from cells was extracted using TRIzol (Invitrogen, USA). qRT-PCR was performed with SYBR Green Master Mix (Thermo Fisher Scientific, USA) in a 7500 Real-Time PCR System (ABI, USA). The expression levels of mRNAs were calculated using the comparative threshold cycle (Ct) method. The primers sequences were as follows: PCMT1 (sense,5′-GG CGTCACAGAGCGCAT-3′; antisense, 5′-GGAG CAGGTACAGAACCACC-3′). GAPDH (sense,5′-TC CTGGTATGACAACGAAT-3′; antisense, 5′-GGTC TCTCTCTTCCTCTT-3′) [44].

### Western blot

After 48 h of transfection in six-well plates, the total protein from cells was extracted using RIPA (Beyotime Biotechnology). The BCA method was used to detect the protein concentration. The same amounts of protein samples (40 μg) were separated by 12% SDS-PAGE, and then transferred to polyvinylidene fluoride (PVDF) membranes. Next, the membranes were blocked with 5% skim milk powder at room temperature for 2 h. The membranes were washed three times with TBST for 10 min each time. Next, the membranes were incubated with primary antibodies overnight at 4°C. The primary antibodies used were as follows: PCMT1 (10519-1-AP, Proteintech, dilution 1:100), AKT (Ab8805, Abcam, Cambridge, UK, dilution 1:500), P-AKT (Thr 308, Ab38449, Abcam, dilution 1:500), GSK-3β (AF5016, Affinity, USA, dilution 1:500), P-GSK-3β (Ser 9, AF2016, Affinity, dilution 1:500), Snail (AF6032, Affinity, dilution 1:500), E-cadherin (Ab76055, Abcam, dilution 1:100), N-cadherin (AF5239, Affinity, dilution 1:500), caspase-3 (Ab184787, Abcam, dilution 1:2000), Bax (AF0120, Affinity, dilution 1:500), and Bcl2 (AF6139, Affinity, dilution 1:500). The membranes were washed twice as described above and incubated with homologous secondary antibodies (Boster, Wuhan, China) at room temperature for 2 h. The membranes were washed as described above. ECL reagent (Applygen, Beijing, China) was added to the membranes and the membranes were placed on an automatic chemiluminescence imaging device (Bio-Rad, USA) to develop. ImageJ software was used to perform grayscale analysis [45].

### Cell proliferation assay

The Cell Counting Kit-8 (CCK-8) assay was performed to detect the cell proliferation ability. Following transfection transfected for 24 h, PC3 and DU145 cells (5000 cells per well) were seeded into 96-well plates, and 100 μl culture medium was added to each well. Next, 10 μl CCK-8 reagent (Biosharp, Hefei, China) was added to each well after culturing at 37°C for 24, 48, 72, and 96 h, and then incubated for 2 h. The absorbance values at 450 nm were measured using a microplate reader (PerkinElmer, USA). In addition, an EdU experiment was also utilized to explore the effect of PCMT1 on the proliferation of PCa cells. Cells in the logarithmic phase of growth were placed in 24-well plates, then incubated with EdU of 50 μM at 37°C for 2 h. After that, cells in each well were fixed with PBS that contains 40% paraformaldehyde, infiltrated with PBS containing 0.5% Triton X-100 and then dyed with Apollo. Finally, the cell nucleus was dyed. Cell proliferation was assessed by counting the proportion of green cells with EdU markers as a percentage of all cells. Finally, colony formation experiment was used for proliferation of PCa cells. 1000 cells were taken from the logarithmic stage and planted in six-well plates, and incubated at a constant temperature for one week. The cell colonies were cleaned with PBS, then fixed with 4% paraformaldehyde and then stained with 0.1% crystal violet. Cell colonies were taken pictures and counted (>50 cells) [46].

### Cell apoptosis assay

After transfecting for 48 h, the Annexin V-FITC/PI apoptosis detection kit (KeyGEN BioTECH, Nanjing, China) was used to detect apoptotic cells by flow cytometry. The apoptosis rate was calculated from the sum of the proportion of early apoptotic cells and late apoptotic cells in the total number of cells.

### Wound-healing assay

A wound-healing assay was performed to detect the migration ability of PCa cells. Cells in the logarithmic growth phase were averagely inoculated into six-well plates, and were transfected after 24 h. Then the cells were routinely cultured with fresh complete medium. When the cells were confluent, a 200 μl pipette tip was used to scratch the surface. The cells were washed twice with PBS, and continued to culture in serum-free medium. The scratches were photographed under an inverted microscope (Olympus, Tokyo, Japan). ImageJ software was used to measure and analyze the scratch area [47].

### Transwell migration and invasion assays

Transwell migration and invasion assays were used to detect the migration and invasion ability of PCa cells respectively. For transwell invasion assay, Matrigel (Corning, USA) was diluted 9 times with serum-free medium and 45 μl diluent was added into each upper chamber of 24-well plates. After a few hours in the incubator, 2 × 10^5^ PC3 cells or 1 × 10^5^ DU145 cells with 200 μl serum-free medium were added to the upper chambers. Next, 700 μL RPMI 1640 medium with 20% fetal bovine serum was added to the lower chamber. After culturing for 24 h, the cells on the upper surface of the chamber were wiped off with a cotton swab. The cells on the bottom surface of the chamber were fixed with 4% paraformaldehyde for 20 min and stained with 0. 1% crystal violet for 20 min. The stained cells on the bottom surface of the chamber were photographed under a microscope. We randomly selected 5 visual fields and used ImageJ software to perform cell counting analysis. The procedures used for the transwell migration assay were similar to those of the transwell invasion assay, except that the upper chambers were covered without Matrigel [47].

### Statistical analysis

Statistical analysis was performed using SPSS 21.0 software. GraphPad Prism 8.0 software was used to generate the graphs. Quantitative data were expressed as $\bar{x}\pm \text{s},$ and comparison between two groups was performed by Student’s *t*-test. χ^2^ test was used to analyze the correlation between the clinicopathological characteristics of patients with PCa and PCMT1 protein expression. *P* value < 0. 05 was considered statistically significant.

### Tumor xenograft formation experiment in nude mice

The animal experiments were approved by the Ethics Committee of Renmin Hospital, Wuhan University (Wuhan, China). Twenty 4-week-old male BALB/c nude mice weighing 18–20 g were fed under specific pathogen free (SPF) conditions (Room temperature: 20 ~ 26°C; Daily temperature difference: ≤4°C; Relative humidity: 40% ~ 70%; Air change times: 15 ~ 20 times/h; Airflow speed: ≤0.2 m/s; Pressure gradient: 20 ~ 50 Pa; Noise: ≤60 dB; Animal illuminance: 15 ~ 20 lx; Alternating time of light and dark: 12/12 h.). After 7 days of adaptation in the laboratory animal facility of Wuhan University People’s Hospital, the mice were randomly divided into vector groups, PCMT1 groups, si-NC groups, si-PCMT1 groups. The cultured 1 × 10^6^ vectors, PCMT1, si-NC, or si-PCMT1 DU145 cells, were diluted with 200 μL serum-free medium and injected into armpits of the forelimbs for tumor formation in nude mice. Tumor formation sizes in mice were measured and recorded every five days. The mice were killed 30 days later, and the tumors were dissected and separated to measure their volume (length × width^2^ × 0.5 mm3) and weight, and photographed. Finally, the tumor tissue was fixed with 4% paraformaldehyde for further experiment and analysis.

### Preprint

A previous version of this manuscript was published as a preprint [48].

### Availability of data and materials

The datasets used during the current study are available from the corresponding author upon reasonable request.
